# Letter from Dr. A. Wadgymar, Corrizo Springs

**Published:** 1886-11

**Authors:** A. Wadgymar

**Affiliations:** Corrizo Springs, Texas


### "TAIT'S OPERATION IN TEXAS."

*By A. Wadgymar, M. D., Corrizo Springs, Texas.*

Case 1, 1884, February 9—Mrs. D. V., age 26, dysmenorrhœa, sterility, successful; child born March 19, 1885.

Case 2, Mrs. B. W., aged 23, catalepsy; cause, amenorrhœa; suppression, of nine years standing.

Case 3, Mrs. G., age 28 years; dysmenorrhœa; all cases cured.

